# 6-mm versus 10-mm covered self-expandable metal stents and plastic stents for malignant distal biliary obstruction in resectable or borderline resectable pancreatic cancer in Japan (STARDOM, JON-2402P): study protocol for a multicenter randomized controlled trial

**DOI:** 10.1186/s13063-026-09821-1

**Published:** 2026-06-13

**Authors:** Daiki Yamashige, Susumu Hijioka, Ichiro Yasuda, Nobuhiko Hayashi, Masataka Taguri, Makoto Ueno, Junji Furuse, Japan Oncology Network in Hepatobiliary and Pancreas

**Affiliations:** 1 Department of Hepatobiliary and Pancreatic Oncology, National Cancer Center Hospital, Tokyo, Japan; 2 Third Department of Internal Medicine, University of Toyama, Toyama, Japan; 3 Department of Health Data Science, Tokyo Medical University, Tokyo, Japan; 4 Department of Gastroenterology, Kanagawa Cancer Center, Yokohama, Japan

**Keywords:** Covered self-expandable metal stent, Distal biliary obstruction, Neoadjuvant chemotherapy, Pancreatic cancer, Plastic stent, Preoperative biliary drainage

## Abstract

**Background:**

Pancreatic cancer is one of the most lethal malignancies; patients with resectable (R) or borderline resectable (BR) disease are treated with neoadjuvant chemotherapy (NAC) followed by surgery. In those with distal biliary obstruction, preoperative biliary drainage enables the timely initiation and completion of NAC followed by surgery. The optimal stent type remains undetermined; 10-mm covered self-expandable metal stents (CSEMS) provide long patency but increase the risk of cholecystitis and pancreatitis, whereas plastic stents (PS) are safer but have shorter patency. A 6-mm CSEMS potentially offers a balanced option that reduces adverse events while maintaining patency during the preoperative period. The STARDOM trial is designed to determine whether 6-mm CSEMS can maintain stent patency while reducing adverse events in preoperative biliary drainage for R/BR pancreatic cancer with distal biliary obstruction.

**Methods:**

In a multicenter, open-label, three-arm superiority randomized controlled trial at 89 institutions across Japan, including academic hospitals and high-volume referral centers, 330 adult patients (≥ 18 years) with R/BR pancreatic cancer and distal biliary obstruction requiring preoperative biliary drainage will be recruited by investigators at each participating institution and randomly assigned (1:1:1) to receive either a 10-mm CSEMS, PS (7Fr or 8.5Fr), or 6-mm CSEMS. Patients with severe acute cholangitis or gastrointestinal obstruction will be excluded. Randomization will be stratified by institution, resectability status, and history of cholecystectomy. The primary endpoint is the event-free survival proportion at 12 weeks, defined as the absence of stent-related events including recurrent biliary obstruction (RBO), pancreatitis, cholecystitis, non-occlusive cholangitis, gastrointestinal bleeding, aspiration pneumonia, liver abscess, perforation, or death. Secondary endpoints include the event incidence rate at 12 weeks, event-free survival proportion, and event incidence rate at 12 and 24 weeks in R and BR patients, respectively; the proportion and incidence rate of each primary endpoint component; technical and clinical success; re-intervention success; and surgical outcomes such as surgical resection proportion, operative time, blood loss, postoperative complications, hospital stay, R0 resection proportion, vascular invasion, and lymph node metastasis.

**Discussion:**

The STARDOM trial is a nationwide multicenter randomized controlled study to directly compare 6-mm CSEMS with conventional 10-mm CSEMS and PS in the pancreatic cancer preoperative setting. Clarifying the optimal drainage method potentially improves perioperative safety, ensures uninterrupted NAC, and supports better oncosurgical outcomes in patients with R/BR pancreatic cancer.

**Trial registration:**

Japan Registry of Clinical Trials (jRCT ID: jRCT1042250093; registered on August 9, 2025). Trial registry URL: https://jrct.mhlw.go.jp/latest-detail/jRCT1042250093. First planned enrollment: August 25, 2025.

**Supplementary Information:**

The online version contains supplementary material available at 10.1186/s13063-026-09821-1.

## Introduction

### Background and rationale {9a}

Pancreatic cancer remains one of the most lethal malignancies worldwide, with a reported 5-year survival rate of approximately 10% in global estimates [1]. Patients with resectable (R) or borderline resectable (BR) disease are increasingly treated with neoadjuvant chemotherapy (NAC) followed by surgery [2–5]. In those with distal biliary obstruction, the appropriate preoperative biliary drainage is needed for the timely initiation and completion of NAC and subsequent surgery. However, stent-related adverse events (AEs), such as cholangitis, obstructive jaundice, cholecystitis, or pancreatitis often occur before surgery and have been reported as risk factors for early postoperative mortality and poor prognosis owing to NAC interruptions [6–10]. Therefore, the more optimal drainage method should provide sufficient stent patency until surgery while minimizing AEs and thereby enable a seamless transition to surgical treatment.

In fact, the 2017 European Society of Gastrointestinal Endoscopy clinical guideline recommends 10-mm metal stents as the standard for malignant distal biliary obstruction [11]. However, despite their long patency, especially 10-mm covered self-expandable metal stents (CSEMS) are reported to cause cholecystitis in 4.2–19.9% and pancreatitis in 7.6–12.1% of patients owing to their non-physiological diameter [12–15]. In contrast, plastic stents (PS) are thinner and generally safer; however, they are prone to recurrent biliary obstruction (RBO), with reported RBO rates of 52.0–72.8% in patients undergoing neoadjuvant chemotherapy, which can lead to cholangitis or jaundice and frequently necessitates re-intervention [9, 16–22]. This is likely because NAC extends the preoperative waiting period, increasing the risk of RBO. Despite this limitation, PS continue to be widely used owing to their safety and ease of placement.

Recently, the 6-mm CSEMS has been reported as a potential compromise. Previous reports show lower rates of cholecystitis and pancreatitis compared with 10-mm CSEMS, and superior stent patency compared with PS without increasing AEs [23–25]. In addition, a phase II prospective study (PURPLE-SIX study) demonstrated a low AE rate of 3.1% (95% CI, 0.0–16.2%) after 6-mm CSEMS placement for malignant distal biliary obstruction in R/BR pancreatic cancer [26]. Considering these findings, 6-mm CSEMS potentially provide an optimal balance between patency until surgery and fewer AEs, both of which are crucial in the preoperative management of pancreatic cancer. In Japan, however, a nationwide survey of 66 institutions (with multiple responses allowed) revealed that 10-mm CSEMS were used in 26 institutions (39%), 7- or 8.5-Fr PS in 37 institutions (56%), and 6-mm CSEMS in 6 institutions (9%) for preoperative biliary drainage in R/BR pancreatic cancer, indicating that the optimal stent choice remains a controversial issue. The STARDOM trial was therefore designed to determine the most appropriate stent for preoperative biliary drainage in this population.

### Explanation for the choice of comparator {9b}

In this trial, the 10-mm CSEMS and PS are used as comparators because they are the current standard devices for preoperative biliary drainage in patients with R or BR pancreatic cancer. The 10-mm CSEMS provides long patency but is associated with higher rates of cholecystitis and pancreatitis, whereas the PS provides safer profiles but is associated with shorter patency.

As the preoperative drainage period is generally limited to a few months, prolonged stent patency is not necessarily required. The 6-mm CSEMS is expected to provide sufficient patency during this period while reducing the risk of procedure-related AEs. Therefore, comparing 6-mm CSEMS with conventional 10-mm CSEMS and PS will clarify the optimal stent selection for preoperative biliary drainage in this population.

### Objectives {10}

The primary objective of this study is to evaluate the superiority of a 6-mm CSEMS in event-free survival proportion at 12 weeks compared with a 10-mm CSEMS and PS in patients with R or BR pancreatic cancer requiring preoperative biliary drainage.

## Methods: patient and public involvement and trial design

### Patient and public involvement {11}

Patients and members of the public were not involved in the design, conduct, reporting, or dissemination plans of this trial. The study was developed by the steering committee based on clinical evidence and an identified unmet need in optimizing preoperative biliary drainage for pancreatic cancer. The results of this trial will be disseminated through peer-reviewed publications and presentations at scientific meetings.

### Trial design {12}

**Fig. 1:**
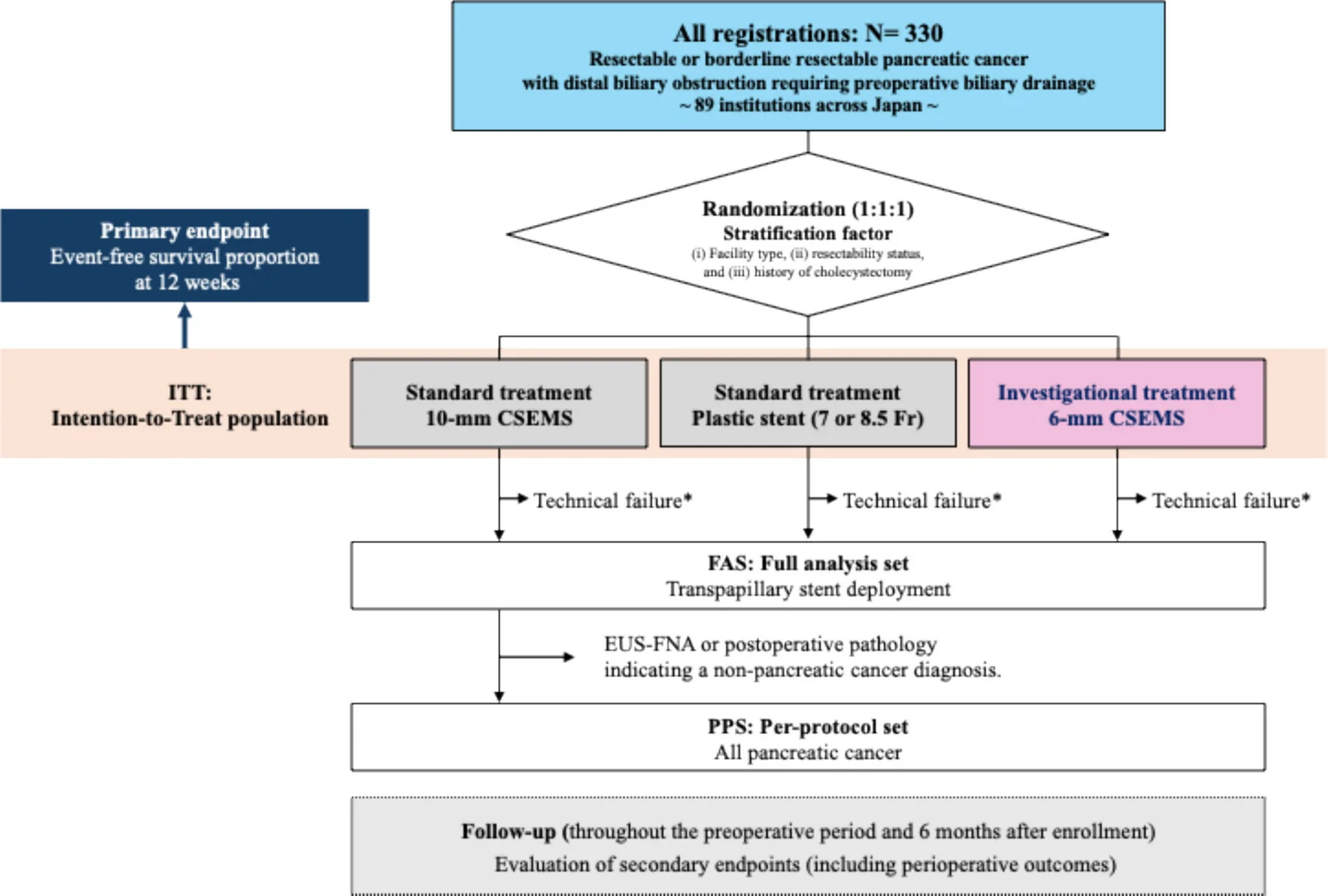
Study flow. *Technical failure: Scope passage impossible due to duodenal stenosis, failed transpapillary cannulation, etc. CSEMS, covered self-expandable metal stent; EUS-FNA, endoscopic ultrasound-guided fine-needle aspiration

This is a prospective, multicenter, open-label, randomized controlled trial conducted in Japan. Eligible patients will be randomly assigned (1:1:1) to receive a 10-mm CSEMS, PS, or a 6-mm CSEMS. The study has been designed with a superiority framework to evaluate whether the 6-mm CSEMS provides better event-free survival at 12 weeks compared with the 10-mm CSEMS and PS (Fig. 1).

## Methods: participants, interventions, and outcomes

### Trial setting {13}

This study will be conducted at 89 institutions across Japan, including academic hospitals and high-volume referral centers specializing in hepatobiliary and pancreatic diseases. The coordinating centers are the National Cancer Center Hospital (Tokyo, Japan) and the University of Toyama (Toyama, Tokyo). Among the 89 institutions, 53 are members of the Japan Oncology Network in Hepatobiliary and Pancreas (JON-HBP). The participating institutions and investigators are listed in Supplementary Table 1.

Characteristics of the people who are needed for the trial:

**Table:** 

Characteristic	The people we would expect to see included
Age	Adults aged ≥ 18 years; expected mean age approximately 65–75 years, reflecting the epidemiology of pancreatic cancer
Sex	Male, female, and intersex individuals are eligible; a slight male predominance is expected
Gender	Individuals identifying as men or women; other gender identities will be reported if collected
Race, ethnicity and ancestry	Majority expected to be East Asian, as the trial is conducted in Japan
Socioeconomic status	No restrictions; participants will be recruited regardless of socioeconomic background
Geographic location	89 centers across Japan, including urban and regional hospitals
Other characteristics relevant to the trial	Resectable or borderline resectable pancreatic cancer with distal malignant biliary obstruction requiring preoperative endoscopic transpapillary biliary drainage and planned neoadjuvant therapy; Eastern Cooperative Oncology Group (ECOG) performance status 0–1

### Eligibility criteria for participants {14a}

#### Inclusion criteria

1. Patients aged ≥ 18 years at the time of consent.
2. ECOG performance status of 0 or 1 (0 = fully active; 1 = restricted in physically strenuous activity but ambulatory).
3. Imaging findings on contrast-enhanced computed tomography (CT) or magnetic resonance imaging (MRI) consistent with invasive pancreatic ductal adenocarcinoma.
4. Diagnosed as R or BR pancreatic cancer on contrast-enhanced CT or MRI, with preoperative therapy planned; the content of the preoperative therapy is not specified, and radiotherapy is also permitted.
5. Presence of distal biliary obstruction (≥ 2 cm distal to the hepatic hilum) requiring biliary drainage, as confirmed by contrast-enhanced CT or MRI.
6. No prior biliary drainage (PS, endoscopic nasobiliary drainage, self-expandable metal stent, percutaneous transhepatic biliary drainage, or percutaneous transhepatic gallbladder drainage) for malignant biliary obstruction; previous drainage for benign disease is allowed.
7. Written informed consent provided by the patient.

#### Exclusion criteria

1. Acute cholangitis of Grade III (severe) according to the Tokyo Guidelines 2018 [27].
2. Presence of gastrointestinal obstruction symptoms.
3. Suspected anomalous pancreaticobiliary junction on imaging (CT, MRI, abdominal ultrasonography, or endoscopic ultrasonography [EUS]).
4. Reconstructed gastrointestinal anatomy other than Billroth I.
5. Ongoing antithrombotic therapy that cannot be discontinued or substituted according to the relevant guidelines [28].
6. Judged as inappropriate for study participation by the investigator.

### Eligibility criteria for sites and those delivering interventions {14b}

All participating centers are capable of performing endoscopic biliary drainage and are approved by the study steering committee.

### Who will take informed consent? {32a}

Informed consent will be obtained by the investigator or a sub-investigator at each participating institution. Prior to enrollment, the investigator will provide a full explanation of the study objectives, procedures, potential risks, and benefits to eligible patients using an IRB-approved consent form. Written informed consent will be obtained from each participant before any study-specific procedures are performed.

### Additional consent provisions for collection and use of participant data and biological specimens {32b}

No additional consent will be obtained for the collection or use of participant data or biological specimens beyond those required for this trial.

All data used in the study will be obtained as part of routine clinical practice or the scheduled study procedures described in this protocol.

This study does not involve the collection or storage of biological specimens for genetic or molecular analysis. No specimens will be retained for future use.

## Intervention and comparator

### Intervention and comparator description {15a}

Eligible patients will be randomly assigned in a 1:1:1 ratio to one of the following three groups:

i. 10-mm CSEMS group: Placement of a 10-mm CSEMS for biliary drainage.
ii. PS group: Placement of a 7-Fr or 8.5-Fr PS for biliary drainage.
iii. 6-mm CSEMS group: Placement of a 6-mm CSEMS for biliary drainage.

All stent placements will be performed via endoscopic retrograde cholangiopancreatography (ERCP) under fluoroscopic guidance. After cannulation of the common bile duct, endoscopic sphincterotomy will be performed in all cases, followed by stent deployment across the papilla. Stent length will be determined based on fluoroscopic measurement of the stricture length. Pancreatic stent placement is allowed only when the pancreatic guidewire method is used. Prophylactic gallbladder drainage is not permitted. No specific devices or stent brands are specified for use in this study. For CSEMS, both fully covered and partially covered types are acceptable. For PS, straight and pigtail stents (single or double) are acceptable.

Concomitant medications such as antibiotics, non-steroidal anti-inflammatory drugs (NSAIDs) for pancreatitis prevention, or hydration therapy are not specified and may be used according to institutional policy. Furthermore, sedation and analgesia are initiated at the discretion of each endoscopist. Antithrombotic management should follow the Japanese guidelines for endoscopy in patients receiving antithrombotic therapy [28]. No scheduled stent exchange will be performed, as stent patency is one component of primary composite endpoints. If stent dysfunction or AEs occur, re-intervention will be performed as needed, and cross-over use of another stent type is permitted after discontinuation of the protocol treatment. Preoperative and postoperative therapies, including neoadjuvant chemotherapy or radiotherapy, are not specified and will be recorded in the case report form (CRF).

### Criteria for discontinuing or modifying allocated intervention/comparator {15b}

Discontinuation or modification of the allocated intervention may occur under the following circumstances:

1. Failure of technical stent placement during the index procedure.
2. Development of procedure-related adverse events requiring alternative management.
3. Recurrent biliary obstruction requiring re-intervention.
4. Withdrawal of consent by the participant.
5. Investigator judgment that continuation of the allocated strategy is not in the participant’s best interest.

All deviations from the allocated intervention will be documented, and all randomized participants will be included in the intention-to-treat analysis.

### Strategies to improve adherence to intervention/comparator {15c}

To ensure adherence to the intervention protocol, all participating endoscopists will receive a standardized explanation of the procedural steps during a pre-study meeting. This meeting will serve to harmonize procedural details. In addition, study investigators at each site will be responsible for ensuring protocol compliance. Central monitoring and regular communication among centers will help identify and resolve any protocol deviations throughout the study period.

### Concomitant care permitted or prohibited during the trial {15d}

Concomitant medications and supportive therapies are not strictly regulated. The use of NSAIDs, antibiotics, hydration, sedatives, and analgesics is at each investigator’s discretion. Antithrombotic management will follow according to the relevant guidelines [28]. The use or non-use of these therapies will be recorded as exploratory data. Neoadjuvant and postoperative therapies, including chemotherapy and radiotherapy, are not restricted and will be documented in the CRF.

### Ancillary and post-trial care {34}

All medical care provided in this study will be covered by the patients’ standard health insurance. Therefore, no additional compensation for medical expenses or special post-trial care will be provided. If any AEs related to the study occur, participants will receive appropriate medical treatment as part of routine clinical care at each participating institution.

### Outcomes {16}

The primary endpoint is the event-free survival proportion at 12 weeks after the endoscopic procedure day. This is a composite endpoint comprising the following events: RBO (requiring re-intervention), pancreatitis, cholecystitis, non-occlusion cholangitis, gastrointestinal bleeding, aspiration pneumonia (within 5 days after the endoscopic procedure), liver abscess, gastrointestinal perforation, or death. Each event is defined according to the Tokyo Criteria 2024 [29], as summarized in Table 1. Only events of moderate severity or higher are included.

**Table 1:**
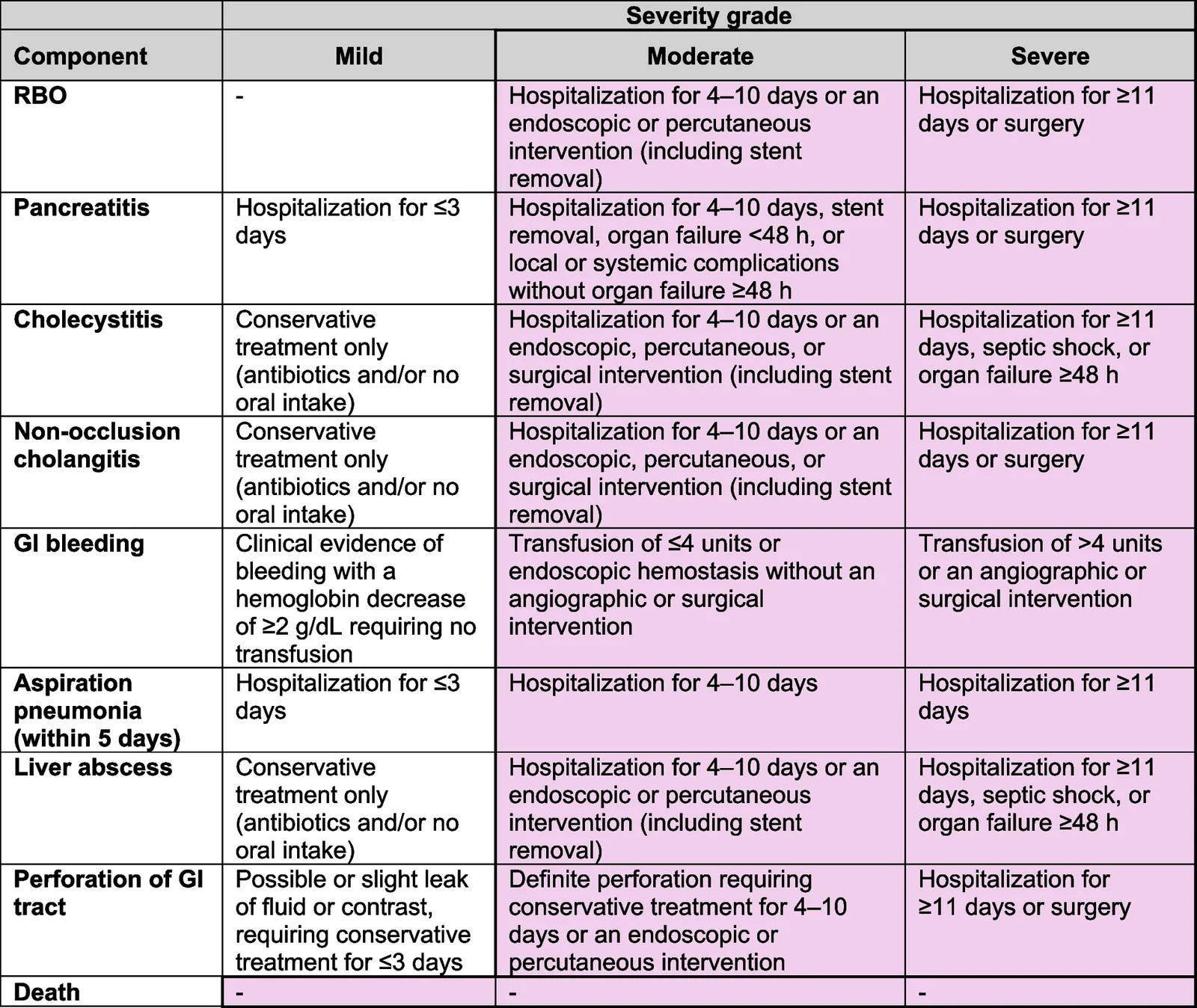
Components and definitions of the primary endpoint

Events corresponding to moderate or severe grade (red color boxes) were defined as endpoint events

*RBO* recurrent biliary obstruction, *GI* gastrointestinal

These events are regarded as treatment failures in the context of preoperative biliary drainage for pancreatic cancer, and the event-free survival proportion at 12 weeks is evaluated as the primary endpoint. When multiple events occur, the earliest one is considered. These outcomes were selected to reflect clinically meaningful treatment failure during the preoperative period, including interruption of neoadjuvant therapy, need for re-intervention, and perioperative complications.

The secondary endpoints include:

1. Event incidence rate at 12 weeks in all patients.
2. Event-free survival proportion and event incidence rate at 12 weeks in resectable cases, and at 24 weeks in borderline resectable cases.
3. The proportion and incidence rate of each component event of the primary endpoint.
4. Technical and clinical success rate according to Tokyo criteria 2024 [29].
5. Successful re-intervention rate, defined as the ability to perform re-stenting in patients who required stent removal due to recurrent biliary obstruction or other AEs.
6. Surgical outcomes, including surgical resection proportion, median operative time, median blood loss, postoperative complications, median hospital stay, R0 resection proportion, vascular invasion, and lymph node metastasis.

### Harms {17}

In this trial, AEs and serious AEs (SAEs) will be collected from enrollment until the end of the protocol-defined follow-up period. AEs will be assessed and documented in accordance with Tokyo Criteria 2024 [29] for procedure-related events and with the Common Terminology Criteria for Adverse Events (CTCAE) version 5.0 for non-procedure-related clinical events. Procedure-related events of interest include pancreatitis, cholecystitis, recurrent biliary obstruction, non-occlusion cholangitis, gastrointestinal bleeding, aspiration pneumonia occurring within 5 days of the procedure, liver abscess, perforation of the gastrointestinal tract, and death. For each AE, investigators will record the date of onset, severity grade, clinical course, and the assessed relationship to the allocated intervention.

SAEs are defined as events that result in death, are life-threatening, require hospitalization or prolongation of existing hospitalization, or lead to persistent or significant disability. SAEs will be reported immediately to the coordinating center in accordance with institutional and regulatory requirements. The coordinating center and the trial steering committee will periodically review accumulated safety data and, when necessary, request further clinical details or evaluation to ensure appropriate safety oversight.

If an unexpected serious AE is judged to be related to the study intervention, the investigator will report the event to the institutional ethics committee in compliance with national regulations. Decisions regarding the need for protocol modification, continuation, suspension, or termination of the trial based on safety concerns will be made by the trial steering committee in consultation with the coordinating center.

The relationship between each AE/SAE and the intervention will be determined by the site investigators based on clinical judgment, temporal relationship to the procedure, and predefined grading criteria.

### Participant timeline {18}

**Fig. 2:**
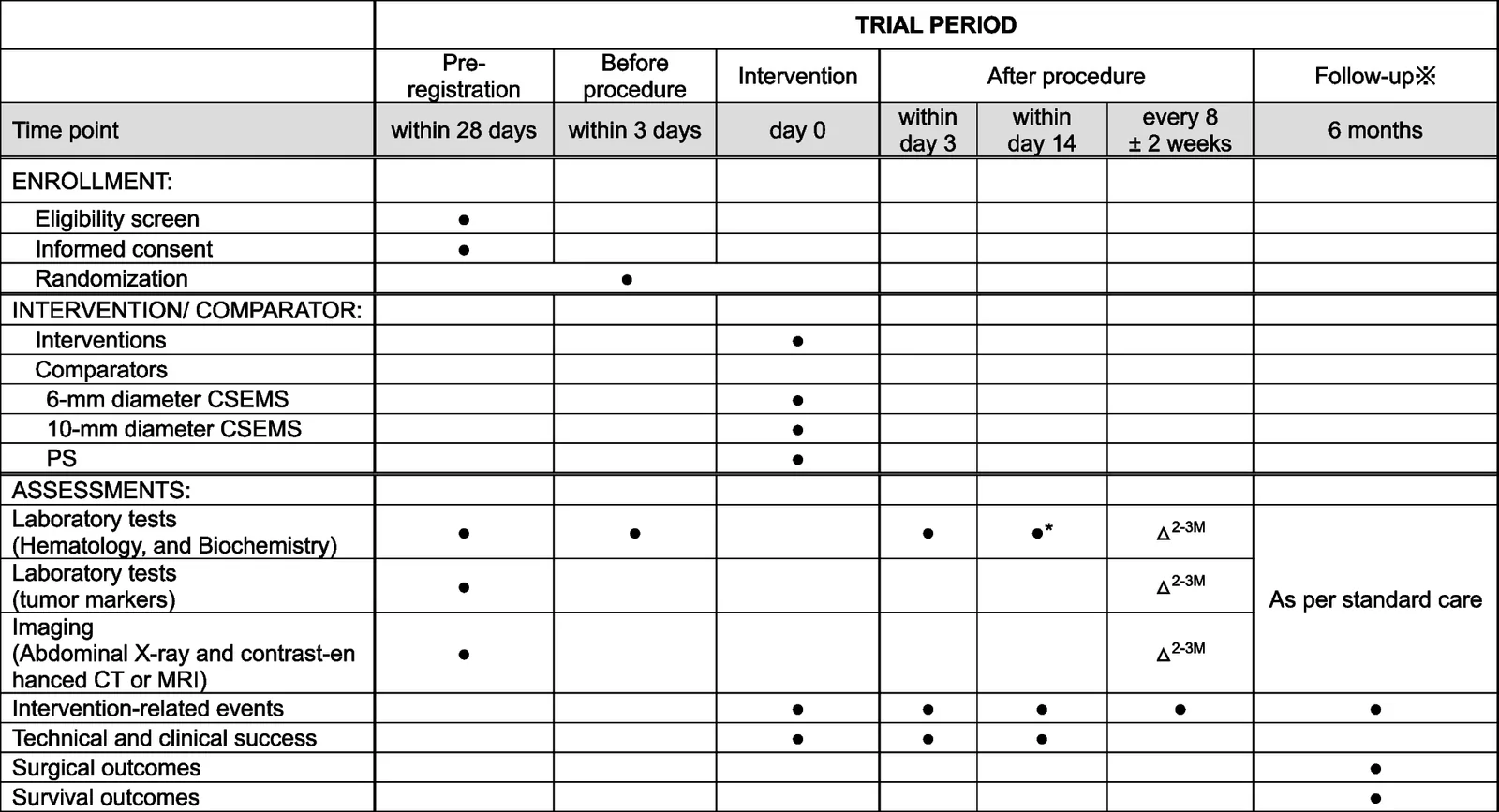
Participant timeline: Schedule of enrollment, interventions, and assessments (SPIRIT figure). *The day 14 laboratory test may be omitted if clinical success is achieved within 14 days. △^2−3 M^: Recommended every 2–3 months (as a guide). ※Follow-up: 6 months from the date of intervention, regardless of surgical resection status. CSEMS, covered self-expandable metal stent; PS, plastic stent; CT, contrast-enhanced computed tomography; MRI, magnetic resonance imaging

Figure 2 shows the participant timeline, including the schedule of enrolment, interventions, follow-up, and outcome assessments throughout the study period.

### Sample size {19}

This study includes two confirmatory superiority hypotheses and one exploratory comparison.

The confirmatory hypotheses are:

i. Superiority of 6-mm CSEMS over 10-mm CSEMS, and
ii. Superiority of 6-mm CSEMS over PS.

The comparison between 10-mm CSEMS and PS is exploratory and will not be used for confirmatory statistical inference.

Based on previous studies in patients receiving neoadjuvant therapy, the 12-week event-free survival proportions were assumed to be 55% for 10-mm CSEMS [18, 22], 50% for PS [18, 23], and 75% for 6-mm CSEMS [26]. The overall one-sided significance level (*α* = 5%) was divided equally between the two confirmatory comparisons using the Bonferroni correction (one-sided *α* = 2.5% for each comparison). With a power of 80% (1–β), the required number of patients per group was calculated to be (i) 98 for the comparison between 6-mm and 10-mm CSEMS, and (ii) 66 for the comparison between 6-mm CSEMS and PS. Since the latter number is smaller, the required number of participants per group was determined as 98. Although the required sample size for the latter comparison (66 cases) is smaller, setting the minimum at 98 patients per group provides higher statistical power and is therefore acceptable. A summary of the statistical power and required sample sizes for each comparison is shown in Table 2.

**Table 2:** Required number of participants according to statistical power

Power	Comparison	
	**10-mm vs. 6-mm CSEMS** **one-sided α = 2.5%**	**PS vs. 6-mm** **one-sided α = 2.5%**
70%	79	53
75%	88	59
80%	98	66
85%	111	74
90%	128	85

Calculations use an overall one-sided α = 5% divided equally between the two primary comparisons by the Bonferroni correction (one-sided α = 2.5% for each comparison: 6-mm CSEMS vs 10-mm CSEMS; 6-mm CSEMS vs PS). Assumed 12-week event-free survival proportions: 55% (10-mm CSEMS), 50% (PS), and 75% (6-mm CSEMS). Randomization is 1:1:1; no interim analysis is planned

*CSEMS* covered self-expandable metal stent, *PS* plastic stent

Assuming a 10% dropout rate, the planned enrollment is 330 patients (110 per group), randomized equally to the three groups using a REDCap-based allocation system. Sample size calculations were performed using R software (version 4.1.2; R Foundation for Statistical Computing, Vienna, Austria).

The planned enrollment period is 3 years, with a 6-month follow-up period after the end of enrollment. 

### Recruitment {20}

Participants will be recruited from 89 institutions across Japan, including 53 high-volume centers belonging to the JON-HBP Group. These institutions routinely manage patients with R or BR pancreatic cancer requiring preoperative biliary drainage; therefore, the planned sample size of 330 patients is expected to be achieved within the 3-year enrollment period without additional recruitment measures.

## Assignment of interventions: randomization

### Sequence generation: who will generate the sequence {21a}

The random allocation sequence will be generated automatically within the REDCap-based randomization module. The system operates independently of investigators responsible for participant enrollment and intervention assignment.

### Sequence generation: type of randomization {21b}

Participants will be randomized in a 1:1:1 ratio to the 10-mm CSEMS group, PS group, or 6-mm CSEMS group using a computer-generated random allocation sequence within a REDCap-based system. Stratified block randomization will be applied, with stratification by facility type (specialized center (cancer center or university hospital) or community hospital), resectability status (R or BR pancreatic cancer), and history of cholecystectomy. The block size will not be disclosed to investigators to maintain allocation unpredictability.　This is an individually randomized trial rather than a cluster randomized trial; therefore, clustering by center was not incorporated into the sample size calculation. Potential institutional heterogeneity is addressed through stratification by facility type.

### Allocation concealment mechanism {22}

The allocation sequence will be centrally stored and automatically managed within the REDCap system. Investigators will not have access to the sequence or future assignments; group allocation will be revealed only after participant registration is completed in REDCap, ensuring allocation concealment until intervention assignment.

### Implementation {23}

The allocation sequence will be generated and maintained by the data center through the REDCap randomization system. Site Principal Investigators or Sub-investigators will enroll eligible participants and enter baseline information required for randomization. After confirmation of eligibility, the REDCap system will automatically assign the participant to one of the three intervention groups. Thus, investigators enrolling participants will not have access to or control over the allocation sequence.

## Assignment of interventions: blinding

### Who will be blinded {24a}

Not applicable; no blinding methods will be used as this is an open-label trial.

### How will be blinding be achieved {24b}

Not applicable; no blinding methods will be used as this is an open-label trial.

### Procedure for unblinding if needed {24c}

Not applicable; no blinding methods will be used as this is an open-label trial.

## Data collection and management

### Plans for assessment and collection of outcomes {25a}

Baseline characteristics, laboratory tests, and imaging findings will be collected before randomization, according to the schedule shown in Fig. 2. Procedural details and outcome events will be collected after randomization and intervention. Data will be collected using standardized electronic case report forms (eCRFs) in the REDCap system. Investigators at each participating institution will be responsible for data entry and outcome assessment according to predefined study definitions and criteria. Data quality will be promoted through centralized data management, query resolution, and periodic data review.

### Plans to promote participant retention and complete follow-up {25b}

Participants will be followed throughout the preoperative period and for 6 months after enrollment. Even if the assigned intervention is discontinued or changed, clinical follow-up will continue, and outcome data (including AEs and re-intervention) will be collected whenever possible.

### Data management {26}

To maintain data integrity, the investigators will ensure that the trial is conducted appropriately, including the accurate generation, recording, and reporting of data. Study data will be entered into the REDCap electronic CRF at each participating site. The data center (The Center for Clinical and Translational Research at Toyama University) will perform central monitoring to check data entry status and consistency and will contact each site as needed to ensure data quality.

### Confidentiality {33}

The Principal Investigator at each site will be responsible for ensuring the confidentiality and secure handling of the participant’s information. Personal identifiers will be stored separately from study data and will not be included in the analysis dataset. All study-related data will be retained in accordance with institutional policies and relevant regulations for the required storage period after study completion.

## Statistical methods

### Statistical methods for primary and secondary outcomes {27a}

Baseline characteristics will be summarized using medians and interquartile ranges for continuous variables and frequencies and proportions for categorical variables. The primary analysis will evaluate whether the 6-mm CSEMS group (C group) demonstrates superiority in 12-week event-free survival compared with the 10-mm CSEMS group (A group) and the PS group (B group). These two comparisons (A vs. C and B vs. C) will be tested as confirmatory analyses, whereas the comparison between A and B will be conducted as exploratory.

Superiority will be judged according to the following four possible outcomes:

- If C is superior to both A and B, C will be concluded to be the sole standard treatment.
- If C is superior to A only, C and B will be considered standard treatments.
- If C is superior to B only, C and A will be considered standard treatments.
- If the superiority of C is not shown in either comparison, A and B will remain standard treatments.

To control the overall one-sided type I error rate at 5%, multiplicity will be adjusted using the Bonferroni method. Accordingly, the A vs. C and B vs. C comparisons will be tested at a one-sided significance level of 2.5%. The A vs. B comparison will be conducted as an exploratory comparison.

The primary analysis will be conducted in the Intention-to-Treat (ITT) population, defined as all randomized participants (Fig. 1). The 12-week event-free survival proportions will be compared using the Cochran–Mantel–Haenszel test stratified by facility type (specialized center [cancer center or university hospital] or community hospital), resectability status (R or BR pancreatic cancer), and prior cholecystectomy. Risk ratios and corresponding 95% confidence intervals will be reported.

Secondary outcomes will be analyzed as follows:

- Event incidence rate will be estimated using person-time methods and compared using Poisson regression.
- Event-free survival curves will be estimated using the Kaplan–Meier method, and between-group comparisons will be performed using Cox proportional hazards models.
- Proportional outcomes (technical success, clinical success, re-intervention success, postoperative complications, R0 resection, vascular invasion, lymph node metastasis) will be summarized using exact binomial confidence intervals. Exploratory comparisons will use Fisher’s exact test.
- Continuous variables (e.g., operative time, blood loss, postoperative hospital stay) will be summarized using descriptive statistics, with exploratory comparisons using the Kruskal–Wallis test.

All statistical analyses will be performed using R software (R Foundation for Statistical Computing, Vienna, Austria) according to the predefined statistical analysis plan. The results of this trial will be reported in accordance with the Consolidated Standards of Reporting Trials (CONSORT) statement. A detailed statistical analysis plan will be finalized before database lock and completion of the primary analysis.

### Who will be included in each analysis {27b}

The primary analysis will follow the intention-to-treat principle and will include all randomized participants, analyzed according to their assigned groups, regardless of treatment received or protocol adherence. Secondary efficacy analyses will be conducted in the full analysis set and per-protocol set, as predefined. Safety analyses will include all participants who underwent stent placement and will be analyzed according to the treatment actually received. Missing outcome data will not be imputed in the primary analysis, and analyses will be based on available cases. Sensitivity analyses using multiple imputation may be performed if appropriate. Detailed handling of missing data will be predefined in the statistical analysis plan.

### How missing data will be handled in the analysis {27c}

Analysis will be conducted in the ITT population, including all randomized participants, and no data will be excluded from the primary analyses solely due to missing values. Cases with missing data will not be excluded; the available data will be analyzed as recorded. In instances of protocol non-adherence, the investigators will document the details and reasons for the deviation and submit them to the Clinical Research Review Committee according to the protocol deviation management procedures.

### Methods for additional analyses (e.g., subgroup analyses) {27d}

Pre-specified subgroup analyses will be performed to explore whether the treatment effect on the primary endpoint differs across clinically relevant categories. The following subgroups will be evaluated:

- Facility type (cancer center/university hospital vs. community hospital)
- Age (< 75 vs. ≥ 75 years)
- Antithrombotic therapy (yes vs. no)
- Resectability status (R or BR pancreatic cancer)
- History of cholecystectomy (yes vs. no)
- Main pancreatic duct dilatation (< 5 mm vs. ≥ 5 mm)
- Cystic duct involvement (yes vs. no)
- Neoadjuvant treatment regimen in resectability status (GS (Gemcitabine plus S-1) vs. GnP (Gemcitabine plus nab-paclitaxel) vs. other)
- Stent type (straight vs. pigtail plastic stent; FCSEMS (Fully CSEMS) vs. PCSEMS (Partially CSEMS); braided vs. laser-cut metal stent)
- Pancreatic duct stenting (yes vs. no)

These subgroup analyses will be exploratory. No adjustment for multiplicity will be performed, and due to potentially limited statistical power and between-group imbalance, results will be interpreted descriptively, rather than for confirmatory inference.

Sensitivity analyses will also be conducted to confirm the robustness of the primary findings:

- Re-analysis of each comparison (A vs. C, B vs. C) in both the FAS and PPS (Per-protocol set) populations using the Cochran–Mantel–Haenszel test, reporting common odds ratios and confidence intervals.
- Fisher’s exact test for categorical comparisons.
- Logistic regression models (unadjusted and adjusted) to estimate odds ratios and confidence intervals.
- In the ITT population, a multivariable logistic regression model will additionally adjust for baseline factors that show imbalance between treatment groups.

All sensitivity and subgroup analyses will be considered exploratory and will not influence the primary conclusions.

### Interim analyses {28b}

No interim analysis for efficacy is planned. However, if approximately half of the planned enrollment (165 patients) is reached and the number of accumulated events is clearly lower than expected (e.g., fewer than 66 events, based on an estimated overall event rate of approximately 40%), a blinded sample size re-estimation may be performed.

No treatment effect estimates will be disclosed during this process.

### Protocol and statistical analysis plan {5}

The full study protocol and statistical code will be made available upon reasonable request to the investigators. Access to de-identified participant-level data may also be considered for academic purposes, subject to approval by the study steering committee and in compliance with applicable data protection regulations.

## Oversight and monitoring

### Composition of the coordinating center and trial steering committee {3d}

The trial steering committee consists of the Principal Investigator (I.Y.), Responsible Investigator (S.H.), Co-Principal Investigators (D.Y., N.H.), and a designated representative investigator from each participating center. The Center for Clinical and Translational Research at Toyama University will serve as the coordinating center, overseeing data management and supporting day-to-day trial operations. The Clinical Research Review Committee will provide oversight and guidance regarding trial conduct, including approval and revision of study documents and authorization of any protocol modifications. In addition to the trial steering committee, an independent efficacy and safety evaluation committee has been established separately from the study investigators. This committee will independently review safety information, study progress, and the appropriateness of trial continuation when necessary.

### Composition of the data monitoring committee, its role and reporting structure {28a}

A Data Monitoring Committee (DMC) will not be established for this study. An independent DMC is not planned because the interventions under investigation—10-mm CSEMS, plastic stents, and 6-mm CSEMS—are all established devices with known safety profiles, and the associated risks are considered manageable within standard clinical practice. In addition, patient monitoring is routinely performed during and after the endoscopic procedure as part of usual care, and the anticipated risk of long-term intervention-related harm is considered low. Therefore, an independent DMC is not deemed necessary.

Instead, safety oversight and data quality management will be conducted through central monitoring by the coordinating center (Center for Clinical and Translational Research, Toyama University) in collaboration with the trial steering committee. The coordinating center will perform regular central monitoring of CRF, safety information, and protocol adherence across participating sites.

The trial steering committee will review AEs, protocol deviations, and study progress throughout the trial. SAEs and clinically important protocol deviations will be promptly reported to the steering committee and discussed among investigators. Corrective actions will be implemented when necessary.

### Frequency and plans for auditing trial conduct {29}

Trial conduct will be monitored centrally by the coordinating center (Center for Clinical and Translational Research, University of Toyama). Central monitoring will include regular review of data completeness, protocol adherence, and consistency of entered data, and clarification will be requested from participating sites when needed. Routine on-site monitoring visits are not planned in accordance with the risk profile of the study and the nature of the intervention. The monitoring process will be conducted independently from the investigators responsible for patient management.

### Protocol amendments {31}

Any important modifications to the study protocol, including changes to eligibility criteria, outcomes, or analytical methods, will be reviewed and approved by the Institutional Review Board or ethics committee before implementation. Following approval, the Principal Investigator (I.Y.), Responsible Investigator (S.H.), Co-Principal Investigators (D.Y., N.H.) will inform all participating investigators and provide updated study documentation. Investigators are responsible for informing participants when changes may affect their continued participation.

### Dissemination policy {8}

The results of the trial, both positive and negative, will be reported at international conferences and/or published in an international journal, without publication restrictions.

## Discussion

This multicenter randomized controlled trial directly compares 6-mm CSEMS with both 10-mm CSEMS and PS in patients with R or BR pancreatic cancer undergoing preoperative biliary drainage. By using 12-week event-free survival as the primary endpoint, the trial is specifically designed to determine whether 6-mm CSEMS can maintain biliary patency and reduce stent-related complications throughout the neoadjuvant treatment period and until surgical resection. If 6-mm CSEMS demonstrate superiority over 10-mm CSEMS or PS, they may be established as the new standard preoperative drainage method. Conversely, if superiority is not demonstrated, the results will still offer high-quality comparative evidence to support individualized stent selection based on clinical context and patient characteristics.

The clinical importance of this question has increased in recent years, as neoadjuvant therapy has become more widely adopted for R and BR pancreatic cancer [2, 3], resulting in a prolonged preoperative period. Safe and effective biliary drainage throughout this extended treatment window is essential to avoid therapy interruption, surgical delays, infection, or nutritional decline. Thus, the findings of this study are expected to have direct relevance to current real-world practice.

In addition, this study will evaluate perioperative surgical outcomes, including resection rate, operative time, blood loss, postoperative complications, hospital stay, R0 resection rate, and other relevant endpoints. Preoperative biliary drainage has been suggested to influence not only drainage-related outcomes but also perioperative complications and long-term prognosis [6–10]. Therefore, assessing perioperative outcomes in this nationwide, 89-center randomized trial provides highly meaningful clinical evidence.

Importantly, biliary stents, unlike anticancer therapies, are frequently introduced into clinical practice without evaluation in large randomized controlled trials, and prior evidence has been limited to observational studies or affected by device-related commercial factors. As an investigator-initiated study, this trial is positioned to generate rigorous evidence that may directly inform clinical guidelines and routine endoscopic practice.

Ultimately, the results of this trial are expected to clarify the role of 6-mm CSEMS in preoperative biliary drainage and contribute to the development of an evidence-based treatment strategy that supports seamless oncological and surgical management in patients with R or BR pancreatic cancer.

## Trial status

Protocol Version 1.6, dated August 09, 2025. Participant recruitment commenced on August 25, 2025 and is currently ongoing. Recruitment is expected to be completed by August 24, 2028, with a 6-month follow-up period after the final participant is enrolled. Final study completion is anticipated on August 24, 2029.

## Supplementary Information

Supplementary Material 1: Supplementary Table S1. Participating institutions and investigators.

## Data Availability

Deidentified individual participant data will not be made publicly available. The full study protocol and statistical analysis plan will be available upon reasonable request from the corresponding author.

## Abbreviations

AE: Adverse event
BR: Borderline resectable
CSEMS: Covered self-expandable metal stent
CTCAE: Common Terminology Criteria for Adverse Events
DMC: Data monitoring committee
FAS: Full analysis set
FCSEMS: Fully covered self-expandable metal stent
GS: Gemcitabine plus S-1
GnP: Gemcitabine plus nab-paclitaxel
IRB: Institutional review board
ITT: Intention-to-treat
PCSEMS: Partially covered self-expandable metal stent
PPS: Per-protocol set
PS: Plastic stent
R: Resectable
RBO: Recurrent biliary obstruction
SAE: Serious adverse event
