# Supplementary material for: 6-mm versus 10-mm covered self-expandable metal stents and plastic stents for malignant distal biliary obstruction in resectable or borderline resectable pancreatic cancer in Japan (STARDOM, JON-2402P): study protocol for a multicenter randomized controlled trial
Source: Trials. 2026 Jun 13;27:539. doi: 10.1186/s13063-026-09821-1 (PMC13488144; doi:10.1186/s13063-026-09821-1)
Supplement: Supplementary file 1 [file 13063_2026_9821_MOESM1_ESM.docx]

**Supplementary table. Participating institutions and investigators**

|  | **Institutions** | **Investigators** |
| --- | --- | --- |
| 1 | Sapporo Medical University | Akio Katanuma |
| 2 | Teine Keijinkai Hospital | Toshifumi Kin |
| 3 | Tonan Hospital | Kei Yane |
| 4 | Hokkaido University | Masaki Kuwatani |
| 5 | Tohoku University | Atsushi Masamune |
| 6 | Fukushima Medical University | Rei Suzuki |
| 7 | Sendai Kousei Hospital | Toru Okuzono |
| 8 | Yamagata Prefectural Central Hospital | Nakao Shirahata |
| 9 | Takasaki General Medical Center | Hidetoshi Yasuoka |
| 10 | Gunma University | Toshio Uraoka |
| 11 | University of Tsukuba | Kiichiro Tsuchiya |
| 12 | Dokkyo Medical University | Atsushi Irisawa |
| 13 | Saitama Medical University International Medical Center | Shomei Ryozawa |
| 14 | Saitama Medical University General Medical Center | Saburo Matsubara |
| 15 | Jichi Medical University Saitama Medical Center | Masanari Sekine |
| 16 | Kameda General Hospital | So Nakaji |
| 17 | National Cancer Center Hospital East | Tomoyuki Satake |
| 18 | Chiba Cancer Center | Emiri Kita |
| 19 | Chiba University | Hiroshi Ohyama |
| 20 | Cancer Institute Hospital, Japanese Foundation for Cancer Research | Masato Ozaka |
| 21 | National Cancer Center Hospital | Susumu Hijioka |
| 22 | Showa Medical University Koto Toyosu Hospital | Jun Ushio |
| 23 | Tokyo Medical University | Takao Itoi |
| 24 | Tokyo Women’s Medical University | Yosuke Nakai |
| 25 | The University of Tokyo | Naminatsu Takahara |
| 26 | Tokyo Metropolitan Tama Medical Center | Hideyuki Horike |
| 27 | Tokyo Metropolitan Bokuto Hospital | Katsumasa Kobayashi |
| 28 | Nihon University Itabashi Hospital | Hirofumi Kogure |
| 29 | Kanagawa Cancer Center | Makoto Ueno |
| 30 | Teikyo University Mizonokuchi Hospital | Shinpei Doi |
| 31 | Tokai University | Eisuke Iwasaki |
| 32 | Yokohama City University Medical Center | Haruo Miwa |
| 33 | Yokohama City University | Kensuke Kubota |
| 34 | JCHO Yamanashi Hospital | Mitsuharu Fukasawa |
| 35 | International University of Health and Welfare Atami Hospital | Yasunari Sakamoto |
| 36 | Shizuoka Cancer Center | Hirotoshi Ishiwatari |
| 37 | Shizuoka General Hospital | Shinya Kawaguchi |
| 38 | Seirei Hamamatsu General Hospital | Yosuke Kobayashi |
| 39 | Hamamatsu University School of Medicine | Kazuhito Kawada |
| 40 | Aichi Medical University | Tadahisa Inoue |
| 41 | Aichi Cancer Center | Kazuo Hara |
| 42 | Nagoya University | Hiroki Kawashima |
| 43 | Fujita Health University | Eizaburo Ohno |
| 44 | Fujita Health University Bantane Hospital | Senju Hashimoto |
| 45 | Mie University | Reiko Yamada |
| 46 | Gifu Prefectural General Medical Center | Kensaku Yoshida |
| 47 | Gifu Municipal Hospital | Keisuke Iwata |
| 48 | Gifu University | Shinya Uemura |
| 49 | Matsunami General Hospital | Mitsuru Okuno |
| 50 | Niigata Cancer Center Hospital | Kazuhiko Shioji |
| 51 | Niigata University | Shuji Terai |
| 52 | University of Toyama | Ichiro Yasuda |
| 53 | Ishikawa Prefectural Central Hospital | Kunihiro Tsuji |
| 54 | Kanazawa University | Hajime Takatori |
| 55 | Shiga University of Medical Science | Takuji Iwashita |
| 56 | Wakayama Medical University | Masayuki Kitano |
| 57 | Kyoto Prefectural University of Medicine | Toshifumi Doi |
| 58 | Osaka Medical and Pharmaceutical University | Takeshi Ogura |
| 59 | Osaka General Medical Center | Takuo Yamai |
| 60 | Osaka International Cancer Institute | Kazuyoshi Ohkawa |
| 61 | Osaka City General Hospital | Satoshi Sugimori |
| 62 | Kansai Medical University | Tsukasa Ikeura |
| 63 | Kansai Medical University Medical Center | Masaaki Shimatani |
| 64 | Kansai Rosai Hospital | Kenji Ikezawa |
| 65 | Kindai University | Mamoru Takenaka |
| 66 | Tane General Hospital | Kotaro Takeshita |
| 67 | Kobe University | Atsuhiro Masuda |
| 68 | Hyogo Medical University | Hideyuki Shiomi |
| 69 | Hyogo Cancer Center | Ikuya Miki |
| 70 | Okayama University | Kazuyuki Matsumoto |
| 71 | JA Onomichi General Hospital | Keiji Hanada |
| 72 | Hiroshima University | Shiro Oka |
| 73 | Yamaguchi University | Shigeyuki Suenaga |
| 74 | Shimane University | Nobuhiko Fukuba |
| 75 | Tottori University | Taro Yamashita |
| 76 | Kagawa University | Hideki Kamada |
| 77 | Shikoku Cancer Center | Akinori Asagi |
| 78 | Matsuyama Red Cross Hospital | Nobuaki Azemoto |
| 79 | Kokura Medical Center | Ken Kawabe |
| 80 | Kokura Memorial Hospital | Tatsuya Noguchi |
| 81 | Kyushu University | Nao Fujimori |
| 82 | University of Occupational and Environmental Health | Shinji Oe |
| 83 | Fukuoka University | Yusuke Ishida |
| 84 | Fukuoka University Chikushi Hospital | Toru Maruo |
| 85 | Oita University | Ryota Sagami |
| 86 | Nagasaki University | Kosuke Takahashi |
| 87 | Miyazaki University | Hiroshi Kawakami |
| 88 | Kagoshima City Hospital | Hiroki Taguchi |
| 89 | University of the Ryukyus | Daiki Agarie |
